# Framing policy objectives in the sustainable development goals: hierarchy, balance, or transformation?

**DOI:** 10.1186/s12992-023-00909-w

**Published:** 2023-01-23

**Authors:** Raphael Lencucha, Alua Kulenova, Anne Marie Thow

**Affiliations:** 1 Faculty of Medicine and Health Sciences, School of Physical and Occupational Therapy, McGill University, Hosmer House, 3654 prom Sir-William-Osler, Montréal, Québec H3G 1Y5 Canada; 2 Faculty of Medicine and Health Sciences, Family Medicine, McGill University, Montreal, Canada; 3 Menzies Centre for Health Policy, School of Public Health, University of Sydney, Sydney, Australia

**Keywords:** Sustainable development goals, Public policy, Framing, Intersectoral, Multisectoral, Health policy, Development

## Abstract

Society continues to be confronted with the deep inadequacies of the current global order. Rampant income inequality between and within countries, dramatic disparities in access to resources, as seen during the COVID pandemic, persistent degradation of the environment, and numerous other problems are tied to existing systems of economy and government. Current global economic systems are implicated in perpetuating these problems. The Sustainable Development Goals (SDGs) were born out of the recognition that dramatic changes were needed to address these intersecting challenges. There is general recognition that transformation of global systems and the relationship between sectors is needed. We conduct a structured, theoretically-informed analysis of SDG documents produced by United Nations agencies with the aim of examining the framing of economic policy goals, a historically dominant domain of consideration in development policy, in relation to health, social and environmental goals. We apply a novel typology to categorize the framing of policy goals. This analysis identified that the formal discourse associated with the SDGs marks a notable change from the pre-SDG development discourse. The ‘transformational’ agenda issued in the SDG documents is in part situated in relation to a critique of previous and existing approaches to development that privilege economic goals over health, social and environmental goals, and position economic policy as the solution to societal concerns. At the same time, we find that there is tension between the aspiration of transformation and an overwhelming focus on economic goals. This work has implications for health governance, where we find that health goals are still often framed as a means to achieve economic policy goals. Health scholars and advocates can draw from our analysis to critically examine how health fits within the transformational development agenda and how sectoral policy goals can move beyond a crude emphasis on economic growth.

**Supplementary Information:**

The online version contains supplementary material available at 10.1186/s12992-023-00909-w.

## Introduction

Society is confronted with deep inadequacies in the current global order. Rampant income inequality between and within countries, dramatic disparities in access to resources, as seen during the COVID pandemic, and persistent degradation of the environment. The dominance of narrowly focused economic policies and institutions and a lack of coherence between social and environmental goals and this dominant economic order can both serve as barriers to addressing health and other goals. Current global economic systems (and corresponding government relationships with these systems) have been largely implicated in perpetuating these problems. The current market system in the absence of government protections has been linked to numerous problems like precarious work [1], the rise and increased power of health harming industries [2, 3], and the general hollowing out of social services [4, 5]. Similarly, the climate crisis has been directly tied to current market systems and more specifically to the ‘economic growth imperative’ [6] that tends to perpetuate overconsumption, overextraction of the natural world, and environment-harming waste [7]. Even mainstream economists have recognized the link between an exclusive emphasis on growth and these ‘negative externalities’ or detrimental ‘spill-overs’ [8]. There are numerous examples of how these problems impact health governance including the reluctance of governments of regulate unhealthy commodity industries in favour of ‘free market’ principles and consumer choice [9]. Despite the known harm, tobacco, unhealthy food, and alcohol companies often justify their products and practices with the rationale that they are contributing to the economy, and governments often echo the same arguments [3, 10–12]. These economic arguments are often received positively by governments and publics despite the known harms to human health and environment perpetuated by these companies. The profit motive was a major factor underlying the unwillingness of pharmaceutical companies to share patent and process information for vaccine development to address the COVID-19 pandemic [13, 14]. Health and environmental protections often confront opposition on economic grounds and within economic legal regimes. Tobacco and food control measures and patent sharing practices have been challenged in trade and investment forums by governments and companies attempting to protect their economic interests [15, 16]. This relationship between economic, health, and environmental goals is tenuous and often conflictual.

Efforts have been made to harmonize these goals and decenter economics in international development plans. The Millennium Development Goals were derived from the United Nations Millennium Declaration adopted by the General Assembly in September 2000, with the broad aim of harnessing the forces of globalization to pursue an equitable development agenda [17]. The MDGs involved eight goals meant to mobilize governments to pursue tangible targets pertaining to poverty, peace and security and environmental protection. Many positive gains were achieved at the end of the MDGs era in 2015 [18]. For example, at the close of the MDGs, reports suggested that global poverty had been cut in half. However, many goals were not met and while aggregate improvements were identified, this period saw an unequal distribution of benefits. Many noted that the aggregate measures of poverty obscured massive increases in income inequality within and between countries and the regional concentration of wealth [19–21]. Similarly, while an aggregate increase in life expectancy indicated some important advances in living conditions globally, these living conditions had not improved universally or evenly. Disaggregated data show persistent disparities in life expectancy within and across regions [22]. In a critique of the Millennium Development Goals (MDGs), Vandemoorele made the point that the emphasis on economic growth and technical (often economic) fixes to societal problems was a major shortfall of the goals, further arguing that achieving sustainable development ‘requires fundamental transformations in society, which transcend macroeconomic, sectoral and institutional models’ [9]. The Sustainable Development Goals (SDGs) were intended to extend and expand the MDGs by more explicitly recognizing the need for ‘transformation’ and ‘coherence’ across policy sectors.

The Sustainable Development Goals (SDGs), adopted in 2015, express an ‘urgent call to action’ to address the numerous challenges facing humanity and the planet. The SDG agenda is halfway to its conclusion (2030) and can provide a window into the state of global consensus on the pressing issues of our time. The way goals are framed within the SDG discourse can shed light on where tensions and conflicts exist in the pursuit of health and other goals. This discourse can also provide insight into where policy is being reimagined to address these pressing issues. The SDGs were born out of the recognition that dramatic changes were needed to address these intersecting challenges. The discourse surrounding the SDGs is driven by notions of ‘transformation’ [23, 24]. Those working in public health noted the potential for transformative and even paradigmatic changes to how the different sectors (e.g., environment, trade, industry, health) relate to each other in the pursuit of health, social and environmental well-being [25]. Across health, environment and other disciplines, scholars and advocates have noted the necessity of finding shared policy goals across different sectors [26, 27].

From a public health perspective, the SDGs set a framework that integrates health across sectors, placing a prominent role for health in development policies and programs [28–31]. At the same time, many have highlighted that inherent contradictions remain between economic goals with those of health, environmental and social well-being. Ruckert and colleagues [32] outline how regional trade agreements pose challenges when it comes to government intervention to ensure health protections in the consumer environment, access to health services, and other equity oriented aims. Meurs and colleagues find similar constraints on public sector spending as a result of conditionalities placed on governments by international financial institutions [33]. Their research in Malawi, Uganda and Tanzania found persistent incoherence between the SDGs and the economic policies of major financial institutions like the International Monetary Fund (IMF). These contradictions and tensions between policies across sectors and the aspiration of cross-sectoral ‘coherence’ remains a critical challenge for equity, sustainability, and health. Much of the conflict across sectors lies deep in the values, ideas and norms that guide policy and programming [34]. The meaning and expression of ‘coherence’ is itself disputed. For some, at least from scholars in the health field, coherence implies a disciplinary emphasis on health-in-all-policies [35, 36]. For others coherence is a reconfiguration of sectoral goals around new paradigms of government, market, and society relations [37, 38]. The challenge at the heart of coherence is the need to establish a common vision for development across sectors [37]. One important role for scholarship in this project of coherence is to examine the ways that the different disciplines and sectors relate to each, both on paper and in practice.

The aim of our analysis is to examine the ways in which economic, health, social and environmental goals are framed in relation to each other in documents produced by United Nations agencies pertaining to the SDGs.

## Methodology

Our research involves a theoretically-informed analysis of all SDG-associated documents published by United Nations agencies between 2012 and 2018. We apply a novel typology to categorize the framing of different goals and how they relate to each other. The typology involves three categories that capture and characterize these relationships: hierarchy, balance, and transformation. The typology was developed iteratively as we sought to identify, categorize, and characterize the relationships described in the documents. This study involved a qualitative documentary analysis. This directed content analysis was informed both by the research question and Bacchi’s theoretical approach to ‘problematizing’ how policy issues are framed [39].

### Document collection

We obtained documents spanning the 3-year lead up to the formal adoption of the SDGs by United Nations member states in 2015, and the subsequent 3 years (i.e., 2012–2018) from the United Nations Knowledge Platform (https://sustainabledevelopment.un.org/post2015/owg), including documents from the Rio + 20 Conference in 2012. We chose this timeframe because it allowed us to see how topics were framed in the lead up to the establishment and adoption of the SDGs, which provides insights into the stage of negotiation and formulation and how ideas were aligned or misaligned across topic areas. The choice to include documents published within the 3 years following the adoption of the SDGs was to identify how conflicting frames were resolved or left in tension with each other. We included documents that were explicitly published on the topic of the SDGs or Millennium Development Goals or development, and were published by a United Nations agency, in order to assess the ways that goals were represented in the formal discussions pertaining to the SDGs. Documents were collected between 2019 and 2021. Our emphasis on United Nations agencies is important as these agencies serve as consensus makers and norm setters and often guide country-level policies and programs. Thirty-seven documents were included for analysis (See Additional file 1: Appendix 1 for document information).

### Analytic approach

The data extracted from the documents were organized into a data extraction form that included the title of publication, year of publication, excerpts that referred to economic policy goals and the relationship between these and other goals. We conducted an initial review of the collected documents to confirm that the documents included content on the MDGs/SDGs/Development. Once this review was completed, we conducted an extensive search of keywords such as several variations of the terms ‘economic’ or ‘economic development’ to find specific excerpts of interest. These excerpts were then added to the extraction form along with the relevant page number to allow for easy reference to the original material. This was particularly important as the excerpts are contextualized by the surrounding paragraphs in collected materials.

The first level of analysis was informed by Carol Bacchi’s WPR, or ‘what’s the problem represented to be?’ framework [39]. Bacchi’s framework is designed to identify and critically analyze how policy ‘problems’ are constructed in relation to proposed policy solutions, with a view to discerning how problems are represented. This framework then moves through implications of (potentially different) representations of the problem. Bacchi notes that ‘the WPR approach argues that policies contain implicit representations of the ‘problems’ they purport to address. These problem representations enact ‘problems’ as particular sorts of problems, thus becoming a crucial part of how governing takes place’ [40]. We drew from the WPR framework to identify and interrogate how problems associated with policy goals and their relation to other domains were framed. We read through document content to thematically group the ways that goals were represented and to characterize the relationships between sectoral goals.

Using the WPR framework we began to identify common themes across documents. AK reviewed the documents and started to identify themes associated with sectoral goals. AK and RL met regularly to review the thematic groupings and discuss how these themes addressed the research question with emphasis on how the framing of goals was related across topics. The interpretation of this content led to the creation of a typology that the authors identified as best representing the ways that economic policy goals were framed in relation to other policy goals. The typology (See Table 1) also drew from the previous work of the lead author who has worked at the intersection of health and other issues such as trade and agriculture. The hierarchy category was developed from the author’s observations of the often-implicit hierarchy advanced in policy discourse that places health and other goals in constant reference to the ‘ultimate’ aim of a strong economy, often in terms of economic growth. The ‘balance’ category was drawn in part from the work of Lang [41] who found that issues of trade and other policy goals were often framed as a balance between economics with other considerations. This idea of ‘balance’ between economic and other goals was further developed in work conducted on the relationship between tobacco control and economic policy in different countries around the world [36, 42]. The last category of transformation emerged in part from the ubiquitous reference to transformation found in the SDG documents and in part from the broader discourse on transformational change. We find this emphasis on transformation different futures captured in different literatures, but particularly in the ‘degrowth’, ‘post-growth’, ‘post-capitalist’, and other movements that attempt to reimagine global economic order with particular emphasis on reconfiguring and reimagining the economic and social order [43, 44]. This emphasis on transformation is also both implicit and explicit in many of the critiques that target current models of market activity associated with environmental, health and social harms [45–47] and drives much of the scholarship in post-colonial and de-colonializing approaches to world order [48, 49]. The assumptions embedded in the ways that economics is framed in relation to health, social, and environmental goals generally involve ideas such as the tradeoff between different parts of society and the economy (and concurrent sacrifices of this tradeoff, whether it is related to the economy or its counterparts), difficulties in balancing economic priorities with the needs of other sectors, and the ability of these different sectors to inherently influence economic policy.

**Table 1:** Typology of relationships between economics and health and social goals

Typology	Description
Hierarchy	Hierarchy implies that economic policy objectives are privileged over other policy concerns, but not always necessarily for the sole pursuit of economic prosperity. Economic goals are positioned as more important than other goals and are often understood in a trickle-down pattern. For example, the popular idea that economic growth is a goal of primary importance, and good health and social well-being within populations follows from a strong economy.
Balance	Balance refers to economic goals positioned alongside, or balanced, with other priorities. For example, the idea that economic growth needs to be pursued with equal consideration and weight on health and social prosperity.
Transformation	Transformation is where the reimagining of economic goals and notions of the economy is encouraged because of a shortcoming of current economic theories and practices.

The data were organized according to the three categories of the typology. We then explored the relationships between the three categories.

## Results

The documents we reviewed covered various issues associated with sustainable development ranging from the provision of health care services to the protection of environmental resources. As can be seen in Additional file 1: Appendix 1, the documents included for analysis were produced by a wide range of UN agencies. We found an overarching recognition of the intersecting nature of the issues being discussed. Generally, the pre-2015 documents reflected an emphasis on the problem of development, similar to the emphasis placed on poverty alleviation in the MDG agenda. There was a marked shift post-2015 to an emphasis on transforming systems and approaches to development. Part of the transformation suggested in the documents related to how the different sectors interact with each other and the need for coordination and harmonization across sectors. The SDGs include explicit reference to policy coherence in Article 17. We found that the post-2015 documents placed particular emphasis on balancing different priorities. Despite the emphasis on balancing priorities, economic policy goals were often given priority or framed as key consideration in health, social, and environmental goals. All the documents placed an emphasis, either implicitly or explicitly, on ‘economic hierarchy’, a perspective that positions economic policy goals as the dominant concern with other goals contributing to this aim. For example, we often found reference to health of populations as a means or contributor to economic growth. We illustrate below how health and other goals were often framed as instrumental to achieving economic goals. Economic growth figured prominently in the documents we also found several open critiques of this emphasis on growth, coupled with calls to transform the economic system. When the status quo was critiqued or calls for transformation made, it was common for economic goals to remain prominent in relation to other goals, but the terms like ‘inclusive’ or ‘equitable’ were added to terms like ‘economic growth’, reflecting attempts to reconfigure ways of viewing economic policy goals. We also found consistent calls to transform the system. Although transformation was often framed in terms of finding a balance between narrow economic growth and other goals, some documents included critiques of the way economic goals had dominated development policy and new visions of economies and their relationships with societies, the environment, education, health and other policy goals.

In the following sections, we describe how economic goals remain a prominent concern in the SDG documents (hierarchy), review the critiques brought forward against this status quo of economic hierarchy, and end by situating calls to balance economic goals with other goals in relation to other calls for transformation. The latter illustrates the struggles that exist in how high-level agencies at the UN seek to operate in the current systems of policy and governance while expressing an aspiration to do things differently for the sake of environmental protection, equity-oriented economics, and health and social well-being.

### Embedded economic hierarchy

We found a strong emphasis on economic policy goals. Other goals were often framed in relation to economic goals. Despite the different mandates of the different agencies, often the goals associated with a particular mandate were framed in relation to economics. For example, we see documents produced by the World Health Organization emphasizing how health is beneficial to the economy or is a sound investment. Despite this hierarchy, many documents added qualifiers to terms like economic growth. Documents published in the lead up to the establishment and adoption of the SDGs frequently referred to ‘inclusive and equitable economic growth’ as a ‘necessary requirement’ for poverty eradication [50] (p. 6). Another document echoes this qualification stating that ‘we recognize that sustained, inclusive and equitable economic growth in developing countries is a key requirement for eradicating poverty and hunger’ [51] (p. 29). To illustrate the hierarchy of goals a World Health Organization document published after 2015 in relation to health and the SDGs, notes that ‘More importantly, the economic growth in many low- and middle-income countries has provided, and will continue to provide, major opportunities for increasing domestic health investments’ [52] (p. 29). Another document that discusses non-communicable diseases (NCDs) in relation to the SDGs, illustrates how the instrumental positioning of health in relation to economics occurs in two directions. First, economic growth is presented as a necessity for health investments. Second, health, and specifically NCDs in this case, are framed in relation to economic growth:

*Loss of productivity linked to NCDs is also significant. It has been estimated that there is a reduction in economic growth of 0.5% for every 10% increase in NCD mortality* [16]*. Losses are cumulative, affect different sectors (including health) and are expressed in terms of direct costs (*e.g. *of diagnosis and treatment, absenteeism and loss of productivity) and also indirect costs, as others may have to replace sick people to cover for some of their activities, adding burden of work or other unmet needs* [53]* (p. 3).*

This excerpt illustrates how health is positioned as necessary for economic prosperity, a common framing in the documents. To further illustrate this point, this same document notes that ‘preventing NCDs makes economic sense’ (p. 3). This framing of health as instrumental for economic growth is seen in other documents where the importance of an issue is justified based on its contribution to economic growth. For example, a document published on the topic of ‘mainstreaming trade’ in the SDG agenda, notes that ‘The opportunities that trade generates for greater economic growth, for improved social development and for reducing poverty are well established. Trade contributes to the realization of the SDGs and, as an enabler, serves as a foundation from which to build national, regional and international policies for sustainable development.’ [54] (p. 9). Another report on urban health presents the same instrumental relationship between health and economic goals, noting that ‘A healthy population is essential for creating economically competitive and inclusive cities’ [55] (p. 6). The WHO also notes that investments in the health and social sectors are a ‘driver of economic growth’ (p. 175). This instrumental framing is most dramatic in the following statement that encourages the framing of health and other issues in economic terms, ‘Growth, while not a panacea for all problems, makes poverty reduction and redistribution policies more acceptable to economic and political elites’ [56] (p. 39). The hierarchy is illustrated in the following proposal submitted during the negotiation of the SDGs suggesting that ‘growth’ should be removed from SDG 8:

*Proposed Goal 8: Ensure full and productive employment and decent work for all, promote sustained, inclusive and sustainable economic, social and human development, within planetary boundaries* [57]*.*

The attempt to replace growth with development within planetary boundaries, an explicit attempt to de-center economic goals in favour of environmental and equity considerations, was not taken up in the final version of SDG 8:

*Goal 8: Promote sustained, inclusive, and sustainable economic growth, full and productive employment and decent work for all.*

### Critiques of economic hierarchy and the status quo

While economic policy goals remain dominant in much of the discourse, there is also a pervasive recognition of the limits of this hierarchy and the approach to economic policy. The emphasis on economic hierarchy was often criticized, noting economic policy and practices have led to significant social and environmental harms. Some of the critiques targeted the expressed limits of the MDGs and a need to reconsider economic approaches to development. The critiques of economic hierarchy and the economic status quo noted an ‘overreliance on economic indicators’ [50] (p. 12) and a corresponding ‘mechanistic association of poverty reduction with economic growth’.

The deeper critique found in the documents targeted an inattention to the ‘root causes’ [50] or ‘structural causes’ [58]. These critiques centered on the contrast between the purported benefits of economic goals and the inequalities and exclusion they produce. This critique is captured most pointedly in the following statement in a document on policy innovations for transformative change where the authors note that ‘key aspects of the economy and wealth creation seem no longer to serve humanity’, going on to critique existing remedial approaches such a public-private partnerships, corporate social responsibility, social impact investment, among others as ‘piecemeal’ and asserting that they do not ‘fundamentally improve well-being, empower vulnerable groups or challenge the drivers of social exclusion and insecurity’ [56] (p. 116). Another document articulated the root causes of the problems embedded in the economic status quo as a ‘by-product of colonialism’ and ‘capitalist extractivism’ with an emphasis on the neglect towards segments of the population including women and indigenous populations [59]. It was noted in one document that ‘From the perspective of poverty eradication, equality and sustainable development, *humanizing the economy* is perhaps the greatest challenge facing the international development community’ (emphasis added) [56] (p. 116).

While these forcible critiques of the economic status quo are advanced, the previous section illustrates that more often the economic hierarchy and status quo was left without critique. These critiques informed the two remaining themes of balance and transformation. As noted earlier, there existed tensions within the documents between a clearly stated hierarchy and calls to place less emphasis on and transform economic goals. The main tensions we found are between a persistent emphasis on the dominance of economic policy goals, coupled with critiques of the ways that these goals have been formulated and consequences of these formulations in relation to health, social and environmental harms. At the same time the ‘transformational’ project was consistently emphasized across the documents, namely the need to reimagine development for sustainability and equity. We found that the most prominent approach towards this transformational agenda was an emphasis on ‘balancing’ goals. Within the theme of balance was an implicit tension between continuing to perpetuate a status quo with its associated harms and reimagining something new.

### Is balance the answer when how to transform is the question?

Throughout the documents there were calls for transformation in the policy goals stemming from the SDGs. A consensus document produced by the United Nations following consultation with a variety of stakeholders summarizes the sentiment as such:

*The thousands of people engaged in the consultation are asking for a global development framework, backed by national policy action, to improve their lives by making people across the world less vulnerable, more empowered and more resilient to change. They want leaders to take action to create the conditions for a more equitable and safer world. They see challenges which persist regardless of economic growth, and they want a forward-looking approach that does not burn through the planet’s resources. Their calls suggest an appetite for transformative change, asking global leaders to surpass the confines of current global consensus* [50]* (p. 21).*

In another document published prior to the adoption of the SDGs it is noted that ‘transformational change’ must challenge ‘the status quo’ and result in a ‘system designed under a different development model’ [60] (p. 4). Several documents addressed the relationship between economic growth and the need for transformation by noting the need for a system based on ‘economic transformation and inclusive growth’ [61] (p. 45), or a ‘radical decoupling of economic growth from natural resource consumption and environmental impacts’ [62] (p. 5). Many of the calls for transformative change returned to a need to tackle ‘root causes’ along different lines. Some of these lines included an emphasis on ‘pro-poor investment and growth’, ‘sustainable food and agriculture’ and ‘social protection programmes’ [63] (p. 6). The discussion was centered on the three key domains framing the SDGs: economy, society, and environment.

We found that alongside calls for transformative change was a language and conceptual structure that reflects an ambiguity of where change will come from and what it will look like. We found more a general reference to principles, which in themselves are potent in articulating a vision of transformation that moves beyond a narrow economic rationality. For example, the following statement presented in the UNEP document makes a dramatic call to move beyond growth for environmental protection:

*Highest priority must now be given to policies and actions that promote and enable radical decoupling of economic growth from natural resource consumption and environmental impacts. Such measures will need to lead to great increases in resource efficiencies of the world’s production systems and increased sustainability in the lifestyles its peoples lead. This requirement is so fundamental that Sustainable Consumption and Production (SCP) has been given both an over-arching status and a specific goal among the 17 SDGs* [62]* (p. 5).*

Similar calls were found in a document published by the United Nations Office on Drugs and Crime (UNODC) where it is noted that transformation will require a deep reconfiguring of the way that systems operate:

*Policy debates that highlight the goal of transformation often ignore the deep-seated changes that are required in economic, social and power relations. Without specific attention to how SDG 16 applies in all dimensions of human life — and not only in relation to targets related to political and legal inclusion — it will be impossible to realize the transformative potential of the SDGs* [64]* (p. 21).*

Despite the critique of using economic growth as a prime indicator for development from some groups and agencies, growth continues to be referenced as a core feature of the SDGs. For example, in one document it is noted that change is required in all three domains (economic, social, environmental) but goes onto to say that sustainable development ‘requires changes in economic structures to promote employment-intensive growth patterns that ensure macroeconomic stability and policy space’ [56] (p. 4). Absent such assertions is also a clear characterization of what this new type of growth should look like. It is not specified how employment-oriented growth aligns with environmental considerations, capital distribution, gender-inclusion, or other health and social goals.

Insights into the nature of transformative change that is represented in the SDG-associated documents comes in part with the emphasis placed on ‘balance’ between the three domains. The three domains are often presented as equivalent concerns in relation to sustainability. For example, we read statements like these that discuss issues, in this case the need to revitalize the agricultural and rural development sectors ‘in an economically, socially and environmentally sustainable manner’ [51] (p. 31). In other sections there is an expressed need to achieve a ‘just balance among economic, social and environmental needs’ [51] (p. 21). In an issue brief on governing the SDGs, it is noted that ‘Global governance institutions need to be able to manage the interlinkages among the three dimensions of sustainable development in such as a way as to secure shared and sustainable prosperity’ [65] (p. 6).

What is not as clear from the documents is how this balance is to be characterized. In one summary document from the Secretary General, it is noted that the environmental domain should be prioritized given the existential challenges being faced in this domain [66]. It was illuminating to see the proposed changes to the text of the SDGs, based on input from a variety of civil society actors, sought to strengthen the transformative language of the provisions. For example, we see that amendments were proposed to move away from the language of economic growth and towards sustainability:

*We recognize that people, of all ages and abilities, are at the centre of sustainable development and, in this regard, we strive for a world that is just, equitable and inclusive, and we commit to work together to promote sustainable and inclusive economic development, social development and environmental protection and thereby to engage and benefit all* [57]* (p. 3) (bolded text are proposed amendments).*

Another proposed amendment sought to refrain the involvement of the private sector according to their impact on human rights, gender equity and social and environmental impacts:

*We also acknowledge that the implementation of sustainable development goals will depend on the active engagement of all stakeholders, including those from the public and private sectors, and civil society, noting that any such engagement must be consistent with human rights and gender equality, and regulated, transparent and accountable for its social and environmental impacts* [57]* (p. 4).*

Agencies often appeal for the need to address critical social and environmental problems created by the current economic systems, while at the same time framing their appeals to change using the core features of these same economic systems, such as economic growth. As noted in an earlier section, the WHO often framed its health objectives as a means to achieve economic outcomes. Similarly, we see this with the United Nations Environment Programme (UNEP) where it is stated:

*What is very likely is that failing to move decisively towards (Sustainable Consumption Patterns) SCP will result in a continuation of the established trade off pattern between the SDG objectives, at the expense of sustainable resource use and the environment, such that resource constraints and environmental changes result in even the social and economic SDGs not being attained in the medium- to long-term, and undoing much development that has been so painstakingly achieved in recent decades* [62]* (p. 39).*

After expressing the gravity of the environmental challenges facing humanity and the dire need to move to sustainable patterns of consumption, the document defends the need for change by noting that such change need not challenge the status quo of competition and economic growth, ‘There is evidence from a number of countries and businesses that pursuing an aggressive SCP agenda need not impact negatively on competitiveness and economic growth.’ This pattern of critiquing the system while appeasing the sensibilities of the current status quo of economic policy was persistent throughout the documents. This tension seems to reflect a portion of the quoted text presented earlier about the need to make policies and approaches ‘more acceptable to economic and political elites’ [56] (p. 39).

## Discussion

Our analysis suggests that the post-2015 development discourse within the United Nations represents a departure from the pre-SDG development discourse. We find the language of ‘transformation’ used in the SDG documents is in part a response to the critique of previous development approaches that privilege economic goals over health, social and environmental goals, or position economic development as the solution to these other societal concerns. Our analysis illustrates that the emphasis on transformation is coupled with the recognition that current economic systems continue to cause severe ecological harms and deepen inequalities between and within countries.

### Moving beyond balance

While the emphasis on transformation is prominent in the documents we analyzed, there remains a tension when articulating what transformation looks like, and how it can be pursued. Despite the language of transformation, there remains an overwhelming focus on economic goals. There seems to be an accommodation between the transformative agenda and this primary focus on economic goals through an agenda of ‘balance’ between different goals. The concept and language of balance has emerged in other domains at the intersection of economic and other goals, often reflecting appeasement or appeal to the dominance of economic goals [36, 41]. Often balance is used as a middle ground between reconfiguring how we do economics (e.g., a de-emphasis on GDP growth or strong regulation of labour markets) and keeping things as they are. In this case, balance is encouraged as a way of placing more emphasis on health, social and environmental goals while maintaining economic goals that emphasize growth and free market practices.

We find some appeals for more radical transformation that decenters economic goals or attempts to reconfigure economic goals based on principles of inclusion and equity. This discourse aligns with efforts by economists to bridge economics with social, environmental, and other concerns. The Economics for Inclusive Prosperity network is one example of an initiative with the expressed aim of providing ‘an overall vision for economic policy that stands as an alternative to the market fundamentalism that is often-and wrongly-identified with economics’ as a discipline [67]. Imaginative strands of transformative economics, like the degrowth movement, call ‘for a different kind of economy altogether: an economy that does not require growth in the first place, and which can deliver justice and well-being even while throughput declines’ [38]. We find that rather than a fringe movement, this move to transformation is part of a broader movement that is reconsidering the limits of the current global order in attending to human well-being, environmental sustainability, and equitable governance. At the same time scholars note that the SDGs remain in contest with the policy of major international financial institutions who have power to shape government mandates and practices [33, 46, 68] and continue to perpetuate aspects of a neoliberal, market fundamentalist agenda (e.g., privatization of public goods like education and health services).

These findings reflect earlier observations of the difficulty in pursuing transformative change in a context where ‘two-track’ thinking is the norm. This two-track thinking involves the recognition of ecological harm, and even crisis, while continuing to advance a system that causes this harm. Leahy and colleagues illustrate how current government policies and industry practices perpetuate ecological harm by using the Australia mining sector as a case example [69]. They note several traps of addressing the climate crisis while perpetuating current modes of production where ‘every individual company and nation is economically compelled to externalise the costs of environmental side effects in order to stay in the capitalist game’ [69]. Our finding support Leahy et al.’s analysis by illustrating that the documents we analyzed put forward a solution of ‘balance’ to the problems that face our world, while at the same time both acknowledging the limits of current economic goals and perpetuating a hierarchy of goals that sees economics sitting at the top. In a similar vein, Hickel argues that the way that the economic policy goal of ‘growth’ is characterized as a neutral, socially uplifting process, obscures a more accurate ‘process of elite accumulation, the commodification of commons, and the appropriation of human labour and natural resources – a process that is quite often colonial in character’ [38]. Rather than embracing the critique of these economic processes, the language of balance says that we just need to pay more attention to the other goals of health, environment, and others. Similar to the tensions between a transformative agenda and the status quo of certain economic policy goals found in our analysis, we see governments struggling to ‘balance’ a friendly corporate environment, including subsidies and other incentives provided to the fossil fuel and other industries [70], with climate change mitigation strategies and other policies oriented to health and well-being [71].

### Engaging with the development agenda for health and sustainability

Economic interests have long relied on economic arguments to oppose, delay, or undermine mitigation policies [72]. This practice of opposing regulatory policy using economic framing is common across sectors. From a health policy perspective, there are numerous recent examples. In the domain of non-communicable disease prevention, there is a long history of industry opposition to public health policy by appealing to the economic mandates of governments and the undesirability of regulation and ‘red tape’ [73]. Much of the discourse in opposition to the regulation of health-harming products like tobacco and unhealthy foods is couched in arguments about preserving jobs and contributing to the economy [11, 12, 74]. Food companies whose core business is the production, distribution and sale of unhealthy products have been able to position themselves as ‘part of the solution’ to the health-related problems that are caused by their practices in part because of the influence over governments, which is tied to their purported contribution to economic goals [75–78]. They have also been able to leverage economic policy instruments such as Trade and Investment Agreements to oppose regulation [79]. Similar examples are found in the fossil fuel sectors, where interests exert significant influence over policy agendas by nefarious practices like the systematic distribution of misinformation, but also through appeals to the entrenched mandates of governments to generate revenue and foster growth [72, 80]. What this body of research has shown is that this form of opposition is simply an ongoing effort by powerful companies to protect their profits [3].

The findings of this study indicate that despite the significant shift in discourse and conceptualizations of ‘development’ that the SDGs represent, there is still a need to shift embedded paradigms regarding what ‘development’ means and how it can be achieved. Two avenues to paradigm-level policy change relevant to our findings include the exercise of power by actors on and within institutions, and policy failure [81]. First, there is potential to disrupt policy paradigms through creation of new institutional structures and coalitions. The creation of the SDGs and associated institutions has been an important first step, but our findings suggest that addressing institutional norms and discourse will be a critical second step. Norms regarding industry-led economic growth are entrenched in Ministries of Economy, Industry and Trade globally, resulting in an openness to industry engagement and a commonality of thinking, because the norms of policy institutions also reinforce a neoliberal economic agenda [2, 82]. Second, the rising global recognition of the failure of ‘traditional’ economic approaches to deliver equitable and inclusive outcomes for the good of society presents an opportunity for a more expansive discourse regarding policy failure in relation to narrow conceptualisations of economic growth and development [67, 83]. The articulation we found for a new vision in response to policy change may be partly explained by the inability of the MDGs to be achieved within the dominant economic (neoliberal) paradigm. There remains an important need to identify where and why current approaches fail to adequately address key objectives such as climate change and non-communicable diseases [84]. At the same time, it will be important to imagine and discuss new ways of bringing different sectors together to address the needs of the age.

## Limitations

This study analysed publicly available documentary data to identify ‘official’ frames and discourse related to development objectives at the global level. Such formal frames can offer important insights about power and influence, in terms of which actors’ interests are represented in policy making [85]. However, this focus on visible outcomes is also a limitation, because – particularly from documentary data – it is hard to impute what underlies these evident inclusions and frames and the ways in which power has been used behind the scenes [86]. The time frame of the study spanned a critical period of change in global development policy, but also precludes detailed study of the long-term historical frames and paradigms that may have generated some of the policy legacies that we see here [87]. This study has highlighted the potential for further research into not only the evolution of the dominant discourse and frames in global development policy, relevant for health, but also research into opportunities for paradigm change.

## Conclusion

The findings of this study speak to the normative tensions that exist in global policy. The transformative agenda of the SDGs is challenged by economic policy objectives that have been shown to perpetuate many of the problems facing the health of population, the social well-being of societies, and the sustainability of the natural environment. By exploring norms and frames related to economic policy objectives at the global level, we have drawn attention to the ongoing centrality of economic concerns to global development policy. Despite the centrality of economic concerns, there is a prominent counter discourse that seeks to reimagine both the substance of economic policy goals for sustainable development and the relationship between economic and other sectors. Scholars and advocates can draw from these counter discourses to enhance attention to the transformative agenda espoused within the SDGs. It is important for health advocates and scholars to critically assess how the framing of policy goals may perpetuate hierarchies in priority and agenda setting, and in some cases perpetuate the dysfunctional elements involved in intersectoral policy including an overemphasis on economic goals that may in fact harm human health, social well-being, and the environment.

## Supplementary Information

**Additional file 1: Appendix A.** Documents included for analysis.

## Data Availability

Analysis tables available upon request.

## Abbreviations

IMF: International Monetary Fund
NCDs: Non-communicable diseases
MDGs: Millienium Development Goals
SDGs: Sustainable Development Goals
UNEP: United Nations Environment Programme
UNODC: United Nations Office on Drugs and Crime
WHO: World Health Organization
WPR: ‘What’s the problem represented to be’ Framework
