# Supplementary material for: Framing policy objectives in the sustainable development goals: hierarchy, balance, or transformation?
Source: Global Health. 2023 Jan 23;19:5. doi: 10.1186/s12992-023-00909-w (PMC9869817; doi:10.1186/s12992-023-00909-w)
Supplement: Supplementary file 1 — Additional file 1: Appendix A. Documents included for analysis. [file 12992_2023_909_MOESM1_ESM.pdf]

Year	Document Title	Associated UN Agency
	**PRE 2015**	
2012	The Future We Want (Rio+20)	Division for Sustainable Development Goals in the Department of Economic and Social Affairs
2012	Initial input of the Secretary-General to the Open Working Group on Sustainable Development Goals	UN General Assembly
2012	UN System Task Team on the Post 2015 UN Development Agenda	UN System Task Team formed by UN Secretary General
2013	TST Issue Brief - Global Governance	UN Technical Support Team
2013	TST Issues Brief - Means of Implementation; Global Partnership for achieving sustainable development	UN Technical Support Team
2013	Mainstreaming of the three dimensions of sustainable development throughout the United Nations system	General Assembly Economic and Social Council
2013	Concluding Remarks of CoChairs OWG6 on SDGs	Open Working Group on SDGs
2013	The Global Conversation Begins - Emerging Views for a New Development Agenda	United Nations Development Grou
2013	Lessons learned from the Commission on Sustainable Development	UN General Assembly
2013	A renewed global partnership for development	UN System Task Team
2013	Global governance and governance of the global commons in the global partnership for development beyond 2015	UN System Task Team
2014	Mainstreaming of the three dimensions of sustainable development throughout the United Nations system	General Assembly Economic and Social Council
2014	Final Compilation of Amendments to Goals and Targets	Open Working Group on SDGs
2014	Progress report of the Open Working Group of the General Assembly on Sustainable Development Goals	Open Working Group on SDGs
2014	TST Conceptual Issues	UN Technical Support Team
2014	FINAL COMPILATION OF AMENDMENTS TO GOALS AND TARGETS	Major groups and stakeholders to inform the OWG on SDGs

Appendix A. Documents included for analysis

Year	Document Title	Associated UN Agency
	**POST 2015**	
2015	UNEP Policy Coherence of the SDGs	United Nations Environment Programme
2015	WHO Health in 2015 - from MDGs to SDGs 2015	World Health Organization
2016	Global Report on Urban Health	World Health Organization
2016	The Sustainable Development Goals are coming to life	United Nations Development Group
2016	Policy Innovations for Transformative Change	United Nations Research Institute for Sustainable Development
2016	NCDs Across the SDGS: A Call for an Integrated Approach	NCD Alliance
2017	FAO and the SDGs Indicators: Measuring up to the 2030 Agenda for Sustainable Development	Food and Agriculture Organization
2017	Guidance Note on Facilitating Integration and Coherence for SDG Implementation	United Nations Development Programme
2017	THE WHO FRAMEWORK CONVENTION ON TOBACCO CONTROL An Accelerator for Sustainable Development: Discussion Paper	World Health Organization
2017	Integration: the key to implementing the Sustainable Development Goals	United Nations University
2017	Fact sheets on sustainable development goals: health targets Noncommunicable Diseases	World Health Organization
2018	FAO Driving action across the 2030 agenda for Sustainable Development	Food and Agriculture Organization
2018	UNISDR Strengthening coherence between climate change adaption and disaster risk adaption	United Nations Office for Disaster Risk Reduction (No longer UNISDR, now UNIDRR)
2018	Mainstreaming trade to attain the Sustainable Development Goals	World Trade Organization, SDGs
2019	ENABLING POLICY AND REGULATION Leaving No One Behind in the Digital Era	UN Capital Development Fund
2019	UNITAR SDGs Main contributions and challenges	United Nations Institute for Training and Research
2019	UNODC Enabling the implementation of the 2030 Agenda through SDG 16+	UN Office on Drugs and Crime
2019	UNODC Tools and Publications Relevant to the SDGs	UN Office on Drugs and Crime
2019	WHO Stronger Collaboration, Better Health - Global Action Plan for Healthy Lives and Wellbeing for All	World Health Organization
